# Effects of 1-methyl-1, 2, 3, 4-tetrahydroisoquinoline on a diabetic neuropathic pain model

**DOI:** 10.3389/fphar.2023.1128496

**Published:** 2023-03-22

**Authors:** Ahmed Tokhi, Zainab Ahmed, Mehreen Arif, Naeem Ur Rehman, Vahid Sheibani, Robert D. E. Sewell, Khalid Rauf

**Affiliations:** ^1^ Department of Pharmacy, COMSATS University Islamabad, Abbottabad, Pakistan; ^2^ Faculty of Pharmacy, Gomal University, Dera Ismail Khan, Pakistan; ^3^ Neuroscience Research Center, Institute of Neuropharmacology, Kerman University of Medical Sciences, Kerman, Iran; ^4^ Cardiff School of Pharmacy and Pharmaceutical Sciences, Cardiff University, Cardiff, United Kingdom

**Keywords:** diabetic neuropathic pain, 1-methyl-1,2,3,4-tetrahydroisoquinoline, hyperalgesia, allodynia, tail immersion test, von frey filaments

## Abstract

**Background:** Neuropathy is a prevalent and debilitating complication of poorly managed diabetes, contributing towards poor quality of life, amputation risk, and increased mortality. The available therapies for diabetic neuropathic pain (DPN) have limitations in terms of efficacy, tolerability and patient compliance. Dysfunction in the peripheral and central monoaminergic system has been evidenced in various types of neuropathic and acute pain. The objective of the present study was to investigate 1-methyl 1, 2, 3, 4-tetrahydroisoquinoline (1MeTIQ), an endogenous amine found in human brain with a known neuroprotective profile, in a model of streptozotocin (STZ) induced neuropathic pain.

**Methods:** Diabetic neuropathy in male BALB/c mice was induced by intraperitoneal injection of a single dose of STZ (200 mg/kg). Upon development of DPN after 4 weeks, mice were investigated for mechanical allodynia (von Frey filament pressure test) and thermal hyperalgesia (tail immersion test). Ondansetron (1.0 mg/kg i.p.), naloxone (3.0 mg/kg i.p.) and yohimbine (2.0 mg/kg i.p.) were used to elucidate the possible mechanism involved. *Postmortem* frontal cortical, striatal and hippocampal tissues were dissected and evaluated for changes in levels of dopamine, noradrenaline and serotonin using High-Performance Liquid Chromatography (HPLC) with UV detection.

**Results:** Acute administration of 1MeTIQ (15–45 mg/kg i.p.) reversed streptozotocin-induced diabetic neuropathic static mechanical allodynia (von Frey filament pressure test) and thermal hyperalgesia (tail immersion test), these outcomes being comparable to standard gabapentin. Furthermore, HPLC analysis revealed that STZ-diabetic mice expressed lower concentrations of serotonin in all three brain regions examined, while dopamine was diminished in the striatum and 1MeTIQ reversed all these neurotransmitter modifications. These findings suggest that the antihyperalgesic/antiallodynic activity of 1MeTIQ may be mediated in part *via* supraspinal opioidergic and monoaminergic modulation since they were naloxone, yohimbine and ondansetron reversible.

**Conclusion:** It was also concluded that acute treatment with 1MeTIQ ameliorated STZ-induced mechanical allodynia and thermal hyperalgesia and restored brain regionally altered serotonin and dopamine concentrations which signify a potential for 1MeTIQ in the management of DPN.

## 1 Introduction

Diabetes mellitus is a serious, metabolic disorder that arises from chronic and persistently elevated levels of blood glucose either due to the body’s inability to produce sufficient insulin, or through resistance offered by body tissues to insulin (IDF, 2021). It is one of the 21st century’s fastest growing health emergencies that has affected an estimated 537 million people in 2021 and may rise to 783 million by 2045. Moreover, some 6.7 million deaths of individuals aged 20–79 have been attributed to diabetes-related complications (Fang et al., 2022; Sun et al., 2022). Diabetic peripheral neuropathy (DPN) is a common and devastating long-term complication in 30%–50% of diabetic patients and it manifests signs and symptoms of peripheral nerve dysfunction. Clinically, detectable DPNs develop within 10 years of the onset of diabetes, and they result in nerve injury initially in large fibers that innervate the feet and subsequently progress proximally (Feldman et al., 2019; Ziegler et al., 2021).

The principal symptoms of DPN include numbness, tingling, sharp pain, and generalized weakness starting in the lower distal extremities with generalized unpleasant sensations (dysesthesia), a painful sensation from normally innocuous stimuli (allodynia), and hyperalgesia that is characterized by heightened pain perception to noxious stimuli (Feldman et al., 2019; Ziegler et al., 2021). Several studies suggest that the management of hyperglycemia and weight control may be insufficient to slow or arrest the progression of DPN (Martin et al., 2006; Boussageon et al., 2011).

Chronic pain experienced in DPN has emotional components with a characteristic expression in the form of anxiety, depression and insomnia (Torta et al., 2017; Ferreira-Chamorro et al., 2018). Over 20% of the population with DPN suffer from major depression and up to a 3-fold elevated level of anxiety (Jain et al., 2011; Huzmeli and Melek, 2017). The increase in disease burden and limitations of current pharmacological treatments arise from their inefficacy, tolerability and adverse effects in addition to associated molecular and neurotransmitter changes related to chronic pain (Fisher et al., 2021). This has prompted the search for newer drug therapies with an improved profile as well as a capability to alleviate the initiation and progression of chronic pain. Despite the prevalence of DPN, currently available centrally acting drug treatments such as antidepressants, anticonvulsants and opioids pose serious challenges by virtue of their frequency of adverse effects, modest efficacy and dependence proclivity (Ang et al., 2018; Fisher et al., 2021).

Monoaminergic systems are not only implicated in central processes including emotion, memory and arousal, but they also play a complex modulatory part in pain signaling (Bannister et al., 2009). Antidepressants are conventionally thought to act *via* monoamines and have been considered as a first-line treatment in chronic DPN. They modulate serotonergic, noradrenergic and dopaminergic systems at least partially in key brain regions involved with pain processing, while atypical opioids also affect monoaminergic activity e.g., tramadol and tapentadol (Mason and Blackburn, 1997). These drugs have focused attention on monoamines and their receptors as potential targets in the development of innovative analgesics (Sindrup et al., 2005; Bannister et al., 2009).

The problem of diabetes mellitus and the variability of clinical outcomes with current therapies is compelling researchers to explore other strategies and potentially effective compounds, some of which are of natural origin, for its treatment and the management of associated complications (Boussageon et al., 2011; Gupta et al., 2021).

1MeTIQ is the methyl derivative of tetrahydroisoquinolines (TIQs), which are endogenous amines present in plants and mammalian brains e.g., humans, monkeys and rodents (Makino et al., 1990; Yamakawa and Ohta, 1997; Yamakawa et al., 1999; Abe et al., 2005). 1MeTIQ possesses neuroprotective, free radical scavenging (Antkiewicz-Michaluk et al., 2006), anti-addictive (Weiss et al., 1992; Antkiewicz-Michaluk et al., 2005; Antkiewicz-Michaluk et al., 2007; Wąsik et al., 2010), antidepressant (Wąsik and Antkiewicz-Michaluk, 2017), anticonvulsant (Pietraszek et al., 2009), anxiolytic and pro-cognitive properties. (Wąsik et al., 2019; Białoń et al., 2020) Moreover, it has been found that 1MeTIQ reversibly inhibits monoamine oxidase A and B (MAO-A and MAO-B *in vitro* and *in vivo* (Patsenka and Antkiewicz-Michaluk, 2004). Furthermore, It has been demonstrated experimentally that 1MeTIQ inhibits not only calcium influx but also 1-methyl-4-phenyl-1,2,3,6-tetrahydropyridine (MPTP) induced parkinsonian-like bradykinesia (Tasaki et al., 1991) and it attenuates the biochemical and behavioral actions of another dopaminergic neurotoxin, rotenone (Antkiewicz-Michaluk et al., 2003). Additionally, it has been documented that 1MeTIQ potentiates morphine-induced analgesia (Wasik et al., 2007), while several other studies indicate that it restores altered monoamine levels in various disease models (Pietraszek et al., 2009; Wąsik et al., 2019). The most significant physiological role proposed for 1MeTIQ is its ability as a natural regulator of the dopamine neurotransmitter system (Rossetti et al., 1992; Antkiewicz-Michaluk et al., 2001; Abe et al., 2005). Considering the diverse neuropharmacological profile of 1MeTIQ, the present aim was to explore any inherent antiallodynic or antihyperalgesic potential in a streptozotocin (STZ) induced *in vivo* model of diabetic neuropathic pain coupled with an evaluation of brain neurotransmitter levels.

## 2 Materials and methods

### 2.1 Experimental animals

Male BALB/c mice weighing 22–26 g were kept at room temperature (22°C ± 2°C) on a 12/12-h light/dark cycle (8:00 a.m. to 8:00 p.m.). They were allowed to habituate for at least 1 week before experiments. Mice had *ad libitum* access to food pellets and water. Animal experimental procedures complied with the United Kingdom Animals (Scientific Procedures) Act 1986 and under the Rules of the Ethical Committee COMSATS University Islamabad, Abbottabad (ethical approval number PHM.Eth/CS-M01-017-1016**)**. Furthermore, it is affirmed that all efforts were made to minimize the number of animals used.

### 2.2 Drugs and chemicals

1-methyl-1, 2, 3, 4-tetrahydroisoquinoline (CAS:4965-09-7) was purchased from Nanjing Dolon Biotechnology Co., Ltd. China, Streptozotocin (CAS: 41910012-3), from Bioshop (Burlington, ON, Canada), Yohimbine from Sigma Aldrich (CAS: 65190). Naloxone 0.4 mg/mL (NALOX^®^ by Rehman Medicines Co.), and Ondansetron 8 mg/4mL (ONSET^®^ by Pharmedic Pvt Ltd.) were purchased locally. Glucometer (One-touch basic blood glucose monitoring system) (Lifescan, Brussels, Belgium) was used.

### 2.3 Induction of the diabetic neuropathic pain model with the STZ-Treatment protocol

STZ was injected as a single dose of 200 mg/kg by the intraperitoneal route (i.p.), freshly prepared in normal saline, and injected within 5 min of preparation (Lynch et al., 1999). Three days after STZ injection, readings of blood glucose levels were evaluated *via* a One-Touch basic blood glucose monitoring system (Lifescan, Brussels, Belgium) and diabetes mellitus was diagnosed if mice had a blood glucose level >250 mg/dL for three consecutive readings (Cheng et al., 2022). They were then housed for 4 weeks to confirm the development of DPN. This animal model reflects human diabetes induced neuropathic pain by generating hyperalgesia and allodynia (Lynch et al., 1999; Kandimalla et al., 2017) and it has been widely employed to mimic neuropathy. The experimenters were not blinded to overall drug treatment since diabetic mice have distinct physical care requirements, for example, the bedding in cages was changed on alternate days to provide dry bedding because animals displayed polyuria. Animals having a heightened pain response to mechanical pressure by means of manual von Frey filament application with a minimal starting pressure (0.008 g) upwards and thermal stimulus (decreased in tail-flick latency) confirmed the onset of the DPN in the model (Zhao et al., 2015; Rehman et al., 2020a; Rehman et al., 2020b). Following STZ injection, a few mice were excluded from the study, as they did not develop diabetes, or they died (6 mice died within 72 h post-STZ administration and 2 expired during experimentation). The dropouts were replaced with another group of mice that had developed diabetes.

### 2.4 Treatment groups of experimental STZ-Induced diabetic mice

Animals were randomly allocated to six experimental groups (n = 6/group) as follows:

Group Ⅰ: saline (vehicle) control, group Ⅱ: (STZ + DPN (positive control), group Ⅲ: standard (STZ + DPN + gabapentin 75 mg/kg), Groups: Ⅳ, Ⅴ, and Ⅵ received different doses of STZ + DPN + 1MeTIQ (15, 30, 45 mg/kg i.p.)

### 2.5 Pain behavioral evaluation

#### 2.5.1 Static mechanical allodynia

The manual von Frey method for assessing mechanical static allodynia is the gold standard procedure for determining mechanical threshold in mice. Animals were placed individually in small cages having a mesh with penetrable base. They were allowed a habituation period of 15–45 min (Su et al., 2020). A series of von Frey filaments (0.008, 0.02, 0.04, 0.07, 0.16, 0.4, 0.6, 1 g), were applied perpendicularly to the plantar surface of the hind paw for 6–8s, in an “up-down” method commencing with a 1 g force, while a 2 g force was selected as a cut-off. A positive response was noted only when the paw was sharply withdrawn, or flinching was presented immediately after von Frey filament application. Ambulation was considered as a false reading and in such cases, the procedure was repeated (Chaplan et al., 1994). Each filament was tested 5 times/paw, and the mechanical threshold (paw withdrawal threshold [PWT]) was defined as the minimal required force that caused at least 3 paw withdrawals observed out of 5 consecutive trials, the result being expressed in gm. A cut-off time of 20 s was imposed to prevent tissue damage (Aman et al., 2016).

#### 2.5.2 Thermal hyperalgesia

In order to quantify thermal hyperalgesia, the mouse tail immersion test was employed whereby a 3.0 cm extremity of the tail was immersed in a water bath thermostatically maintained at 54°C ± 0.5°C. The time (s) between the appliance of the nociceptive stimulus (hot water) and tail-flick was recorded digitally. Testing was performed at 30, 60, 90, and 120 min after treatments, employing a cut-off time of 15 s (Deuis et al., 2017).

### 2.6 Investigation of the possible mechanism involved

In the present study, after revealing the antiallodynic and antihyperalgesic potential of 1MeTIQ in STZ-induced diabetic mice, another investigation was conducted to explore possible underlying pharmacological mechanisms involved using the serotonin receptor (5-HT_3_) antagonist, ondansetron (1.0 mg/kg i.p.); non-selective opioid receptor antagonist, naloxone (3.0 mg/kg i.p.) or an α_2-_adrenoreceptor antagonist, yohimbine (2.0 mg/kg i.p.) for possible antihyperalgesic effects. Animals then received 1MeTIQ (15 mg/kg i.p.) 30 min after the administration of ondansetron, naloxone, or yohimbine and subsequently tested in behavioral experiments 30 min later (Pandurangan et al., 2014).

### 2.7 Neurotransmitter quantification using HPLC

#### 2.7.1 Sample preparation

After behavioral experimentation, mice were euthanized by cervical dislocation. Frontal cortical, striatal and hippocampal tissues were dissected and separated on ice-chilled plates, then precisely weighed (ng/mg of the wet weight of tissue) and stored at −80°C. A Teflon-glass homogenizer (Ultra-Turax^®^T-50) was used for tissue homogenization in 0.2% ice-cold perchloric acid. The sample was then cold centrifuged at 12,000 rpm (4°C) (DLAB Scientific). Subsequently, the supernatant was filtered using a 0.45 mm filter (CNW Technologies). It was then placed for analysis in an HPLC auto-sampler (Rehman et al., 2020a; Rehman et al., 2020b).

#### 2.7.2 Standard preparation

For the preparation of the standard stock solutions, 1.0 mg of either dopamine, serotonin, or noradrenaline were dissolved in 10 mL HPLC grade water. The stock solution of each neurotransmitter was then diluted to make different concentrations of 100–00 ng/mL which was used for the calibration curve. Samples were then placed in an HPLC auto-sampler and 20 µL volume was withdrawn for injection using the software (Empower^TM^). The calibration curve was then constructed by plotting the peak area of dopamine, serotonin or noradrenaline (y) against the concentration of standard dopamine, serotonin or noradrenaline (x) respectively, using linear regression analysis (Rehman et al., 2020a; Rehman et al., 2020b).

#### 2.7.3 Chromatographic analysis

Chromatography was performed using a Waters Alliance e2695 separation module with 2998 PDA UV detector and auto-sampler (United States). A C18 stainless steel column (250 × 4.6 mm) (waters X Select^®^ HSS Ireland) with 5 µm particle size was employed. The mobile phase was comprised of methanol: HPLC grade water (DAEJUNG; Korea: 8585–2304) in a ratio (5:95 v/v) with added 20 mM monobasic sodium phosphate (DAEJUNG; Korea: CAS: 7558–80-7) as a buffer. The UV detection wavelength was 280 nm with isocratic elution. The elution rate was set at a flow rate of 0.5 mL/min and a column temperature of 35°C (Hou et al., 2019; Rehman et al., 2020a; Rehman et al., 2020b).

### 2.8 Statistical analysis

The paw withdrawal threshold (PWT) determined as the minimal force of von Frey filaments (over the range 0.008–1.0 g), were classified as discrete variables in statistical terms. Therefore, the PWT data was presented as box-and-whisker plots with upper and lower quartiles. The tail-flick latency (thermal hyperalgesia) data were presented as mean ± S.E.M. All data was processed using GraphPad Prism version 8. The parameters were examined for normality with the Shapiro-Wilk normality test. One-way ANOVA was used for the analysis of variance and *post hoc* Tukey’s test was employed to analyze the data. *p*-values <0.05 were taken as significant throughout.

## 3 Results

### 3.1.1 Effect of 1MeTIQ on STZ-DPN static mechanical allodynia

**FIGURE 1:**
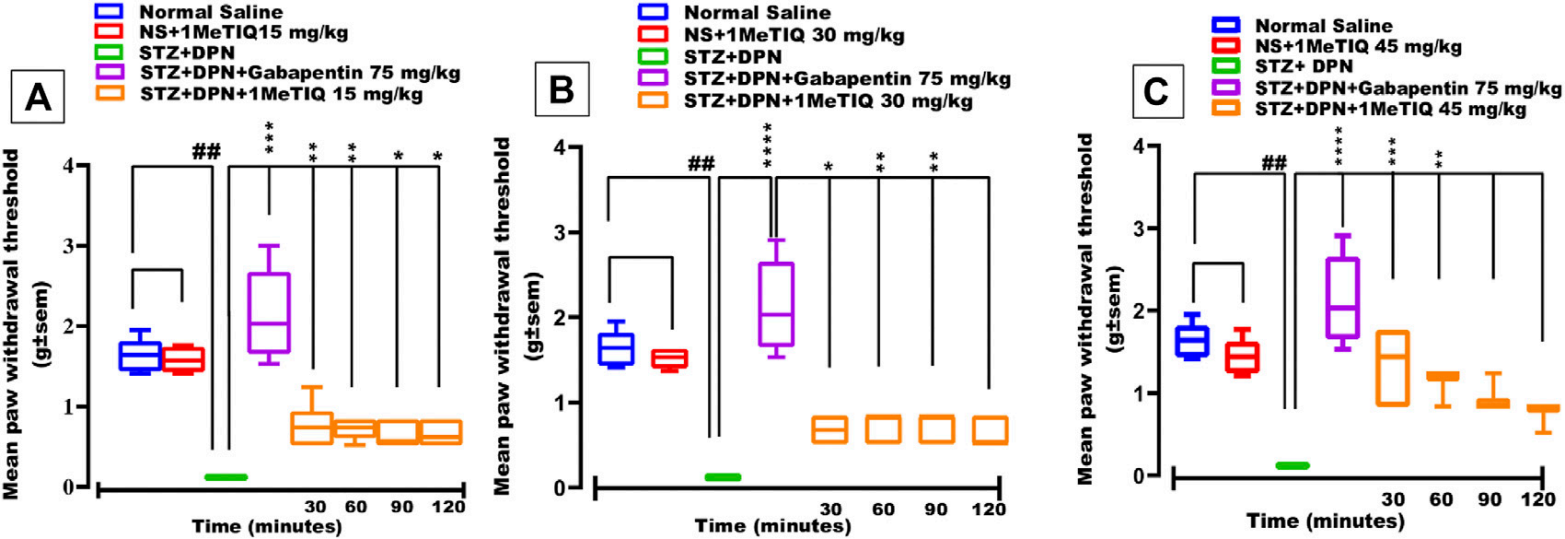
Effect of 1-Methyl-1,2,3,4-tetrahydroisoquinoline (15, 30 and 45 mg/kg i.p.) or gabapentin (75 mg/kg) on STZ-induced diabetic static mechanical allodynia (von Frey filament applied paw withdrawal pressure; PWT). Four weeks after STZ administration, mice displayed markedly decreased responses to nociception and they also became allodynic **(A, B)** and **(C)**, an effect that was overcome by both gabapentin and 1MeTIQ. Data were presented as mean ± SEM and the abscissa coordinate represents the time after treatment. *p < 0.05, **p < 0.01, ****p < 0.0001 when compared to the STZ + DPN group.

Intraperitoneal administration of a single STZ dose produced marked hyperalgesia within a period of 2 weeks and tactile allodynia in 4 weeks with a progressive sensory loss, a key feature that bears a close resemblance to the development of stimulus-evoked pain corresponding to allodynia or hyperalgesia in humans. In animals subjected to the streptozotocin protocol (STZ-DPN), there was a highly significant (*p* < 0.0001) reduction (95%) in PWT (>0.12 g i.e., static mechanical allodynia) which was restored to a level that was comparable to the saline-treated group (>2.5 g) by the dose of gabapentin (75 mg/kg) as the positive control standard drug (Figures 1A–C). Similarly, 1MeTIQ (15 and 30 mg/kg i.p.) administered to animals with streptozotocin-induced DPN produced a marked increase in PWT (>0.7 g) between 30–120 min after dosing, which was suggestive of a restorative action against DPN allodynia (Figures 1A–C). The highest dose of 1MeTIQ (45 mg/kg) induced an even more pronounced reversal of DPN allodynia 30 min after administration (i.e., PWT <1.4 g), to a level not significantly different (*p* < 0.05) from that produced in saline-treated animals. This activity progressively declined with post-treatment time but still maintained statistical significance up to the 60 min post dose exposure time (Figure 1C).

#### 3.1.2 Effect of 1MeTIQ on STZ-DPN induced thermal hyperalgesia

**FIGURE 2:**
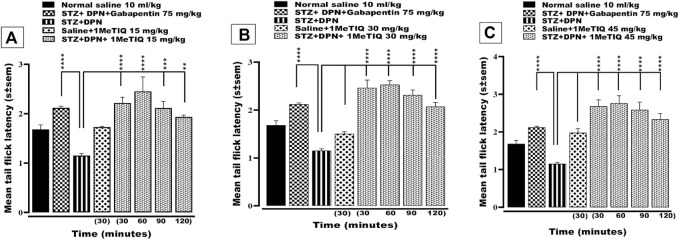
Effect of 1-Methyl-1, 2, 3, 4-tetrahydroisoquinoline (15, 30 and 45 mg/kg i.p.) or gabapentin (75 mg/kg) on STZ-induced diabetic thermal hyperalgesia. 4 weeks after STZ administration, mice exhibited significantly reduced response latencies in the tail immersion test. **(A, B)** and **(C)**, an effect that was negated by both gabapentin and 1MeTIQ. Data are presented as mean ± SEM and the abscissa coordinate represents the time after treatment. **p < 0.01, ***p < 0.001, ****p < 0.0001 when compared to the STZ + DPN group.

Exposure of peripheral sensory nerve endings to elevated temperature above a certain threshold induces a nociceptive sensation and regarding this, the mouse tail immersion test can expose either thermal nociception or hyperalgesia. In the current study, animals that underwent the STZ protocol to induce DPN, exhibited a highly significant statistical reduction (*p* < 0.0001; i.e., decrease by 68%) in tail-flick latency to <1.5 s (thermal hyperalgesia). This hyperalgesic response was counteracted (*p* < 0.0001) by gabapentin as the positive control drug to a level (>2.2 s) that was essentially higher than saline-vehicle treatment (Figures 2A–C). Likewise, DPN-induced thermal hyperalgesia was also reversed by 1MeTIQ (15, 30 and 45 mg/kg i.p.). Thus, all three doses of 1MeTIQ elevated thermal response latencies throughout the whole time-course of the test period up to 120 min with a peak latency at 60 min (>2.4 s), indicating an antihyperalgesic effect. In the case of the two higher 1MeTIQ doses this enhancement was highly significant (*p* < 0.0001) for up to at least 2 hours (Figures 2A–C).

#### 3.1.3 Effect of ondansetron, naloxone or yohimbine on the antihyperalgesic effect of 1MeTIQ against STZ-DPN induced thermal hyperalgesia

**FIGURE 3:**
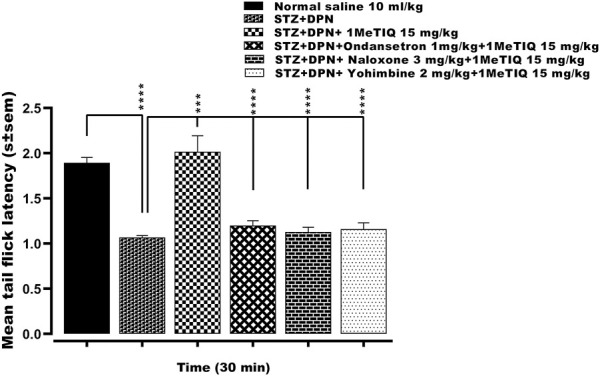
Effect of ondansetron, naloxone or yohimbine administration on the antihyperalgesic action induced by 1-Methyl-1, 2, 3, 4-tetrahydroisoquinoline (STZ + DPN+1MeTIQ 15 mg/kg) against streptozotocin-diabetic peripheral neuropathy (STZ + DPN) induced thermal hyperalgesia (Tail immersion test). Data are presented as mean ± SEM and the abscissa coordinate represents the tail flick latency 30 min after treatment. ***p < 0.001; ****p < 0.0001.

There was a highly significant reduction (*p*˂0.0001, 56%) in the mean tail immersion nociceptive response latency to <1.1s in the animal group with STZ-diabetic peripheral neuropathy-induced hyperalgesia compared to controls. This streptozotocin hyperalgesia was then reversed by 1MeTIQ (15 mg/kg) to a level that was not statistically different (*p* < 0.05) from controls (i.e., >2.1 s). Subsequently, administration of either ondansetron, naloxone or yohimbine each successively abolished the antihyperalgesic activity of 1MeTIQ on streptozotocin hyperalgesia i.e., by a decrease of 88%, 94% or 92% respectively in each case to <1.2 s (Figure 3).

#### 3.1.4 Effect of 1MeTIQ on frontal cortical tissue levels of noradrenaline and serotonin in mice after the STZ protocol

A decrease (43%) in frontal cortical serotonin levels and an increase in noradrenaline concentration (48%) was observed in the animal group that underwent the STZ + DPN protocol. However, treatment of these animals with 1MeTIQ only at the 45 mg/kg dose significantly boosted (*p* < 0.05) frontal cortical levels of serotonin and noradrenaline by 96% and 32% respectively in this group, while gabapentin (75 mg/kg) only raised the noradrenaline concentration as summarized in Table 1.

**TABLE 1:** Concentrations of neurotransmitters in frontal cortex tissue after the streptozotocin protocol (i.e., STZ + DPN) alone or combined with gabapentin or 1MeTIQ. **Note:** *p < 0.05, **p < 0.01.

Treatments	Noradrenaline ng/mg wet weight tissue	Serotonin ng/mg wet weight tissue
Normal saline	19.05 ± 3.96	16.10 ± 3.3
STZ + DPN	39.61 ± 6.96*	7.021 ± 0.99*
STZ + DPN + Gabapentin 75 mg/kg	57.91 ± 15.66**	10.98 ± 1.25
STZ + DPN+1MeTIQ 15 mg/kg	39.77 ± 7.52	12.09 ± 2.17
STZ + DPN+1MeTIQ 30 mg/kg	44.19 ± 6.41	12.09 ± 1.61
STZ + DPN+1MeTIQ 45 mg/kg	59.76 ± 8.39*	15.41 ± 1.14*

#### 3.1.5 Effect of 1MeTIQ on striatal tissue levels of dopamine, noradrenaline and serotonin in mice after the STZ protocol

A significant decrease in striatal levels of dopamine (43%) and serotonin (23%) was detected in the STZ-DPN group (*p* < 0.001) compared to the saline vehicle-treated controls. However, after treatment with (1MeTIQ 15 mg/kg), there were marked increases in concentrations of dopamine (36%) and serotonin (36%), while 1MeTIQ (30 mg/kg) [32%], 45 mg/kg (33%), and gabapentin (75 mg/kg; 73%) significantly raised noradrenaline levels compared to the STZ + DPN group as shown in Table 2.

**TABLE 2:** Concentrations of neurotransmitters in striatal tissue after the streptozotocin protocol (i.e., STZ + DPN) alone or in combination with either gabapentin or 1MeTIQ. **Note:** *p < 0.05, **p < 0.01, ***p < 0.001.

Treatments	Dopamine ng/mg wet weight tissue	Noradrenaline ng/mg wet weight tissue	Serotonin ng/mg wet weight tissue
Normal saline	6.37 ± 0.74	6.559 ± 0.58	1.79 ± 0.19
STZ + DPN	2.74 ± 0.45***	10.20 ± 0.58	0.417 ± 0.6***
STZ + DPN + Gabapentin (75 mg/kg)	3.32 ± 0.45	13.89 ± 1.80	1.23 ± 0.13**
STZ + DPN+1MeTIQ 15 mg/kg	7.60 ± 0.83***	13.58 ± 0.86	1.10 ± 0.88**
STZ + DPN+1MeTIQ 30 mg/kg	5.09 ± 0.38*	16.54 ± 1.49**	1.28 ± 0.15***
STZ + DPN+1MeTIQ 45 mg/kg	5.31 ± 0.27*	16.69 ± 0.66**	1.27 ± 0.10***

#### 3.1.6 Effect of 1MeTIQ on hippocampal tissue levels of noradrenaline and serotonin in mice after the STZ protocol

Hippocampal concentrations of noradrenaline were not modified in the animal group that was subjected to the STZ + DPN protocol, but serotonin levels were suppressed (21%). Nevertheless, treatment of this group with 1MeTIQ at the two higher doses, significantly increased noradrenaline levels (53% and 60%, *p* < 0.05) while all three 1MeTIQ doses raised serotonin concentrations (28, 29 or 25%) in the hippocampus but gabapentin (75 mg/kg) only raised the level of serotonin (22%), as presented in Table 3.

**TABLE 3:** Concentrations of neurotransmitters in hippocampal tissue after the streptozotocin protocol (i.e., STZ + DPN) alone or in combination with gabapentin or 1MeTIQ. **Note:** *p < 0.05, **p < 0.01, ***p < 0.001.

Treatments	Noradrenaline ng/mg wet weight tissue	Serotonin ng/mg wet weight tissue
Normal saline	3.13 ± 0.23	1.3 ± 0.08
STZ + DPN	4.06 ± 0.48	0.27 ± 0.03***
STZ + DPN + Gabapentin 75 mg/kg	6.16 ± 1.31	1.20 ± 0.20***
STZ + DPN+1MeTIQ 15 mg/kg	5.61 ± 0.68	0.96 ± 0.10**
STZ + DPN+1MeTIQ 30 mg/kg	7.65 ± 0.49*	0.94 ± 0.12**
STZ + DPN+1MeTIQ 45 mg/kg	6.79 ± 0.61*	1.08 ± 0.08***

## 4 Discussion

Diabetes has attained pandemic proportions with over half a billion sufferers globally and a health expenditure approaching approximately one trillion US dollars (IDF, 2021). The development of neuropathies are relatively common among diabetic patients (Feldman et al., 2019; Gupta et al., 2021) resulting in lifetime disabilities, poor quality of life, immunosuppression, a decline in cognition, devastating limb amputations, maladaptive stress responses, anxiety, depression, altered sleep patterns and premature deaths (Tesfaye, 2011). The pathogenesis of DPN is multifactorial and hyperglycemia is critically instrumental in contributing to the development and expression of neuropathic pain (Said, 2007; Shillo et al., 2019). In the present study, STZ was employed to induce diabetes in mice (STZ-DPN), resulting in hyperalgesia and allodynia over a 4-week period. Acute administration of 1MeTIQ to STZ-DPN mice increased their paw withdrawal thresholds and elevated their tail flick latencies. These behavioral effects were also corroborated by the neurotransmitter studies *via* HPLC analysis.

In relation to the present study, pancreatic islet β-cells in male mice are more prone to STZ-incited cytotoxicity because of the presence of higher testosterone concentrations and low estrogen levels. In contrast, female mice are more resistant to STZ-induced diabetes due to a protective action of estrogen. However, increasing the dose of STZ can overcome this resistance in female mice though toxicity and lethality are more likely to ensue (Like and Rossini, 1976; Le May et al., 2006). Consequently, arising from this gender difference, male mice were preferentially selected for our investigation.

Tetrahydroisoquinolines (TIQs) represent the simplest group of the non-catechol chemical family, with distribution both in the plant and animal kingdom (Lorenc-Koci, 2012). The methyl derivative i.e., 1-methyl-1, 2, 3, 4-tetrahydroisoquinoline (1MeTIQ) has a special position as it is formed endogenously in the human brain and shares many properties with similar neuroprotectants including free radical scavenging activity (Patsenka and Antkiewicz-Michaluk, 2004), inhibition of MAO-A and MAO-B (Antkiewicz-Michaluk et al., 2001; Wąsik et al., 2014), and inhibition of Ca^2+^ influx with similar neuroprotectants (Antkiewicz-Michaluk et al., 2006).

In mice with streptozotocin provoked DPN (STZ + DPN), mechanical allodynia was produced, which was manifested by a marked decrease in paw withdrawal threshold to von Frey filament application on the plantar paw surface (Figure 1). Likewise, STZ + DPN animals displayed a considerable decrement in response latency in the tail immersion test signifying thermal hyperalgesia (Figure 2).

The mechanical allodynia was alleviated by gabapentin and notably by all doses of 1MeTIQ for a period up to 2 h (Figure 1). In addition, the thermal hyperalgesia was completely restored to control levels over the 2-h test phase by gabapentin and the three doses of 1MeTIQ (Figure 1).

One plausible mechanism for the antiallodynic activity of 1MeTIQ may be *via* an interaction with spinal 5-HT_3_ receptors since extensive evidence has shown that 5-HT_3_ receptors are involved in both the expression and control of mechanical allodynia (Oatway et al., 2004; Bravo-Hernández et al., 2012; Nasirinezhad et al., 2016). It is also appropriate to point out that in the tail immersion test, the effects of 1MeTIQ were comparable to those of the positive control drug, gabapentin. This would imply that the tetrahydroisoquinoline derivative invokes a supraspinal mechanism in brain areas, important to pain perception/processing. Such regions include the frontal cortex that perceives pain signals and generally receives connections from the neocortex, hippocampus, periaqueductal grey (PAG), amygdala, hypothalamus and striatum (Ong et al., 2019).

In order to gain some insight into any possible underlying antinociceptive mechanism, ondansetron (5-HT_3_ antagonist), yohimbine (α2-adrenoceptor antagonist) or naloxone (non-selective opioid antagonist) were administered as pretreatments to STZ + DPN followed by combination with 1MeTIQ (Figure 3). All three mechanistic interactants significantly reversed 1MeTIQ induced antihyperalgesia thus implicating a possible involvement of serotoninergic, noradrenergic and opioidergic mechanisms (Figure 3). These findings are in accordance with a published study where α_2_-adrenoceptors are thought to be engaged in STZ diabetic neuropathy (Buerkle and Yaksh, 1998; Bujalska et al., 2008). Furthermore, ondansetron attenuated 1MeTIQ antihyperalgesia and in this regard, 5-HT_3_ receptors have been reported to exacerbate neuropathic pain in a chronic pain state such as DPN (Silva et al., 2016). Ondansetron has also been reported to reverse tramadol and acetaminophen analgesia in the clinic (Vale et al., 2011; Koyuncu et al., 2017), and because 1MeTIQ antihyperalgesia was reduced by naloxone, it may also be postulated to interact simultaneously with both opioidergic and monoaminergic pathways in generating a thermal antihyperalgesic action. In addition, 1MeTIQ produced an upsurge in serotonin concentration following its suppression by streptozotocin neuropathy in the frontal cortex, hippocampus and striatum. It is relevant to mention at this point, that the central serotonergic system has been implicated in chronic neuropathic pain and a variety of serotonin modulators are in clinical use for its management *via* an enhancement of serotonergic tone both centrally and peripherally (Adamo et al., 2021). The increased central level of serotonin after streptozotocin neuropathy should also be viewed in the context of an inverse duality of 5-HT receptor functions in descending spinal serotonergic systems (de Kort et al., 2021).

Dopamine was only identified above the assay detection limits in the striatal tissue of saline vehicle-treated animals. However, in the STZ + DPN group, there was a marked decrease in striatal concentrations of dopamine which were then reversed by all doses of MeTIQ (Table 2). Over the years, dopamine has been a primary focus of addictive and reward/motivational studies, however, in recent times, its role in pain modulation has gained considerable attention (Mitsi and Zachariou, 2016). Central dopaminergic neurotransmission plays a pivotal role in the modulation of pain and both preclinical and clinical studies indicate that dopaminergic neurotransmission is disturbed during chronic neuropathic pain such as that linked to diabetes (Hagelberg et al., 2004; Mitsi and Zachariou, 2016; Pérez-Taboada et al., 2020). Furthermore, dopamine may also participate in descending pain inhibition, and disrupted dopaminergic tone may heighten pain perception while drugs that facilitate dopaminergic transmission may well provide pain relief (Mitsi and Zachariou, 2016; Serafini et al., 2020). In earlier studies, 1MeTIQ has been documented to have a neuromodulatory effect on dopaminergic neurotransmission (Antkiewicz-Michaluk et al., 2003), and there is evidence that it stimulates dopamine release (Ishiwata et al., 2001; Antkiewicz-Michaluk et al., 2014). In this connection, we found that all three doses of 1MeTIQ under study, restored dopamine levels which had been depleted by induced neuropathy in the striatum of streptozotocin-diabetic mice. What is more, in experimental and clinical studies, a decrease in nigrostriatal dopamine level has been attributed to noxious thermal pain (Chudler et al., 1993; Saadé et al., 1997; Chudler, 1998) and mechanical stimuli (Schultz and Romo, 1987). Moreover, electrolytic destruction of the substantia nigra as well as both 6-hydroxydopamine and kainic acid lesions in the nigrostriatal dopaminergic pathway, tend to increase nociceptive sensitivity. In contrast, electrical stimulation of the substantia nigra and intraventricular administration of apomorphine, or discrete microinjection into the caudate-putamen complex, decrease pain sensitivity (Lin et al., 1981; Saadé et al., 1997). In a slightly more recent clinical study, it has been shown that L-DOPA treatment of Parkinson’s disease patients elevates pain threshold (Brefel-Courbon et al., 2005) and drugs of abuse like amphetamine, cocaine or cannabinoids also mediate antinociception by augmenting dopamine concentrations centrally (Shyu et al., 1992; Altier and Stewart, 1999).

In our study, STZ-induced diabetic mice had significantly raised levels of noradrenaline in the frontal cortex and they were subsequently boosted by 1MeTIQ. This finding is in accordance with that of Możdżeń and coworkers, whereby administration of 1MeTIQ extensively increased noradrenaline and serotonin both in the frontal cortex and striatum in a murine model of depression (Możdżeń et al., 2017). In this respect, it is noteworthy that both of these pathways meaningfully provide a substrate for the drugs that control and mitigate neuropathic pain (Stamford, 1995; Pertovaara, 2006; Caraci et al., 2019).

As a final point, it is evident that STZ-diabetic mice expressed lower concentrations of serotonin in all three brain areas studied, and dopamine was diminished in the striatum while 1MeTIQ reversed each of these neurotransmitter modifications. It is therefore evident that the STZ-induced diabetic state modified neurotransmitter function in the striatum, prefrontal cortex and hippocampus, and that 1MeTIQ limited these diabetic effects in the specific and discrete brain regions examined.

## 5 Conclusion

In summary, we have found that 1MeTIQ has quantifiable antihyperalgesic and antiallodynic effects in streptozotocin-diabetic neuropathic mice. Furthermore, acute treatment with 1MeTIQ restored the neurotransmitter levels that were decreased in these neuropathic animals. Ondansetron, naloxone or yohimbine all abolished the antihyperalgesic action of 1METIQ. So, it can be postulated that 1MeTIQ generates antihyperalgesic and antiallodynic activity at least partially through interaction with central, opioidergic, serotonergic and noradrenergic monoaminergic pathways by means of augmented neurotransmitter tone. Such actions may designate 1MeTIQ as a good candidate to be explored at the cellular level as a novel agent for the management of diabetic neuropathic pain.

## Data Availability

The original contributions presented in the study are included in the article/supplementary material, further inquiries can be directed to the corresponding author.

## Abbreviations

DPN, Diabetic peripheral neuropathic pain; STZ, Streptozotocin; 1MeTIQ, 1-Methyl-1,2,3,4-Tetrahydroisoquinoline; HPLC, High-Performance Liquid Chromatography.
